# Differential interaction of Apolipoprotein-E isoforms with insulin receptors modulates brain insulin signaling in mutant human amyloid precursor protein transgenic mice

**DOI:** 10.1038/srep13842

**Published:** 2015-09-08

**Authors:** Elizabeth S Chan, Christopher Chen, Gregory M Cole, Boon-Seng Wong

**Affiliations:** 1 Departments of Physiology Yong Loo Lin School of Medicine, National University of Singapore, Singapore; 2 Departments of Physiology and Pharmacology, Yong Loo Lin School of Medicine, National University of Singapore, Singapore; 3 Memory Ageing and Cognition Centre (MACC), National University Health System (NUHS), Singapore; 4 Departments of Medicine and Neurology, University of California, Los Angeles, USA.; 5 Geriatric Research and Clinical Center, Greater Los Angeles Veterans Affairs Healthcare System, Veterans Affairs Medical Center, Los Angeles, California, USA

## Abstract

It is unclear how human apolipoprotein E4 (ApoE4) increases the risk for Alzheimer’s disease (AD). Although Aβ levels can lead to insulin signaling impairment, these experiments were done in the absence of human ApoE. To examine ApoE role, we crossed the human ApoE-targeted replacement mice with mutant human amyloid precursor protein (APP) mice. In 26 week old mice with lower Aβ levels, the expression and phosphorylation of insulin signaling proteins remained comparable among APP, ApoE3xAPP and ApoE4xAPP mouse brains. When the mice aged to 78 weeks, these proteins were markedly reduced in APP and ApoE4xAPP mouse brains. While Aβ can bind to insulin receptor, how ApoE isoforms modulate this interaction remains unknown. Here, we showed that ApoE3 had greater association with insulin receptor as compared to ApoE4, regardless of Aβ42 concentration. In contrast, ApoE4 bound more Aβ42 with increasing peptide levels. Using primary hippocampal neurons, we showed that ApoE3 and ApoE4 neurons are equally sensitive to physiological levels of insulin. However, in the presence of Aβ42, insulin failed to elicit a downstream response only in ApoE4 hippocampal neurons. Taken together, our data show that ApoE genotypes can modulate this Aβ-mediated insulin signaling impairment.

Alzheimer’s Disease (AD) is a neurodegenerative disorder characterized by the progressive deterioration of memory and other cognitive faculties, ultimately leading to dementia^1^. The pathological hallmarks of AD include the presence of extracellular amyloid plaques and intracellular neurofibrillary tangles of hyperphosphorylated tau. While AD remains a multi-faceted disease, the human apolipoprotein E4 (ApoE4) allele was identified to be the strongest genetic risk factor for sporadic AD cases, where its expression accelerates the onset of the disease in a gene-dose dependent manner^2^,^3^.

In the brain, ApoE can be produced by both glia and neurons under physiological and pathological conditions, and its classical role is to redistribute lipids among cells in the brain^3^,^4^. ApoE exists in three isoforms, ApoE2 (cys112, cys158), ApoE3 (cys112, arg158) and ApoE4 (arg158, arg158). The ApoE3 genotype occurs in ~80% of most populations; whilst ApoE4 and ApoE2 occurs in ~14% and <7% respectively. By contrast, ApoE4 occurs in >50% in AD^4^. The presence of a cysteine or arginine residue at positions 112 or 158 profoundly changes the tertiary structure and functionality of the protein^5^. Unlike the other two isoforms, ApoE4 exists in a “molten globule” formation, which makes it more prone to proteolytic cleavage. This hinders the protein from carrying out its regular functions and may also result in the formation of neurotoxic ApoE4 fragments^6^.

As it is widely believed that the development of AD pathology begins decades before the emergence of symptoms and substantial neurodegeneration, it is important to understand the disease at its earliest possible stage^1^. Interestingly, the AD brain has been described to be in an “insulin resistant” state, while aberrant insulin signaling has been identified as an early risk factor for the disease^7^,^8^. In postmortem brain samples, insulin and insulin receptors were markedly reduced in postmortem AD brains, and insulin sensitivity was impaired in the hippocampal formation of AD samples^9^,^10^. These data indicate that insulin sensitivity is associated with AD, at least at the end stage.

Insulin is taken up by the brain from pancreatic sources but also produced by neurons in the brain^11^. Insulin is crucial for maintaining neuronal survival, differentiation, metabolism and learning and memory^7^,^8^,^12^. Although insulin receptors are found throughout the brain, they are concentrated in the frontal cortex, hippocampus, amygdala and the olfactory bulb^12^. The insulin signaling pathway is initiated by the binding of insulin to the extracellular subunit of the insulin receptor (IRα). This results in the autophosphorylation of the intracellular subunit of the insulin receptor (IRβ), providing docking sites for the immediate downstream effector, insulin receptor substrate 1 (IRS-1). Recruitment of IRS-1 propagates the signaling cascade, with IRS-1 tyrosine phosphorylation leading to PI3K p85 docking and the activation of kinases such as Akt^12^,^13^. The activation of Akt promotes neuronal survival, and is known to contribute to long-term potentiation (LTP) and CREB phosphorylation at serine residue 133 (S133), which are relevant to memory consolidation^12^,^13^.

Clinical data have shown that increased insulin administration improved the performance of elderly individuals in learning and memory tasks, and performance is correlated to lowered levels of plasma APP^14^,^15^,^16^. These insulin treatment effects are ApoE isoforms dependent; ApoE4 carriers showed no improvement in memory tasks and no reduction to plasma APP levels^15^,^17^. In addition, reports have shown that in the presence of Aβ, ApoE targeted therapy, anti-diabetic agents or insulin sensitivity itself can reduce the load of amyloid plaques or lead to LTP formation^18^,^19^,^20^. Taken together, these data suggest that the insulin sensitivity is changed in the presence of amyloid pathology, and such modulation could be ApoE isoform dependent. However, how ApoE isoforms differentially modulate insulin sensitivity in the presence Aβ is still unknown.

To examine the connection between ApoE isoforms and brain insulin signaling in AD, we created a preclinical mouse model by crossing human ApoE-targeted replacement mice^21^,^22^ with mice carrying familial-AD (FAD) mutant human amyloid precursor protein (APP)^23^,^24^. This mutant APP bears the Swedish (K670N/M671L) and the Indiana (V717F) mutations (APPSwdInd). In this study, we examined both ApoE3 and ApoE4, since these isoforms occur in ~94% of most populations and are represented in >99% of sporadic AD^25^,^26^.

Studies have reported significant cognitive impairment in female ApoE4 mice as compared to male ApoE4 mice^27^,^28^,^29^,^30^. Female mice carrying mutant APP were also observed to bear heavier amyloid burden and display more severe behavioral deficits^31^,^32^,^33^. In AD, ApoE4 was reported to confer greater risk in women^34^. We therefore decided to examine insulin signaling in the brain of female APP, ApoE3xAPP and ApoE4xAPP mice.

## Results

### Aβ content in the aging ApoE-APP mice.

**Figure 1:**
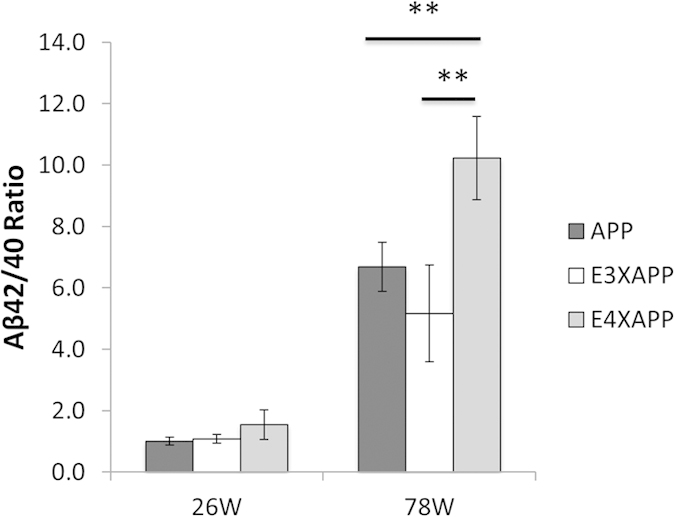
Aβ42/40 ratio in the ageing ApoExAPP mice. Aβ42/40 ratio in the brain of 26 and 78 weeks old APP (dark grey), ApoE3xAPP (E3XAPP, white) and ApoE4xAPP (E4XAPP, grey) mice. Higher Aβ42/40 ratio was detected in the brain of 78 weeks ApoE4xAPP mice as compared to age-matched ApoE3xAPP and APP mice. Each value represents the mean ± SEM for individual mouse brain sample (26 weeks n = 5, 78 weeks n = 7). One-way ANOVA revealed differences between groups (**p < 0.01).

Aβ42 is the predominant species of Aβ in the AD brain and this peptide concentration will increase more than other Aβ species as AD progresses^35^,^36^,^37^. At 26 weeks, there was no significant difference in the Aβ42/Aβ40 ratio (Fig. 1) between APP, ApoE3xAPP and ApoE4xAPP mice. However, at 78 weeks, post-hoc analysis Tukey-Kramer analysis showed that ApoE4xAPP mice had significantly higher Aβ42/ Aβ40 ratio than APP and ApoE3xAPP mice (Fig. 1).

As compared to APP mice, the Aβ42/ Aβ40 ratio in ApoE4xAPP mice is 35% higher. This Aβ42/ Aβ40 ratio in ApoE4xAPP mice was >50% higher than in ApoE3xAPP mice. These results suggested that changes in Aβ burden are ApoE isoforms dependent (one-way ANOVA, F = 11.39, p < 0.01), consistent with other ApoExAPP mouse lines^28^,^38^,^39^,^40^,^41^.

We then examined ApoE expression in the three mouse lines using an antibody specific for human ApoE. In figure S1, no detectable human ApoE expression was detected in the young and old APP mice as this mouse line expresses mouse ApoE. Human ApoE expression was only detected in ApoE3xAPP and ApoE4xAPP mice. The two detected ApoE bands are the native 34 kDa protein and the higher molecular weight sialylated ApoE isoprotein^42^. These bands can be observed in human ApoE transgenic mouse and in human brain, but not in mice expressing mouse ApoE^42^.

### Brain insulin signaling in ApoE-APP mice.

At 26 weeks, there was no difference in the expression and phosphorylation of insulin receptor substrate 1 (IRS-1) and Akt between APP, ApoE3xAPP and ApoE4xAPP mouse brain (Figure S2) (One-way ANOVA, P-IRS-1, F = 0.42; Y608, IRS-1, F = 0.55; P-Akt S473, F = 0.14 and P-Akt T308, F = 0.58).

**Figure 2:**
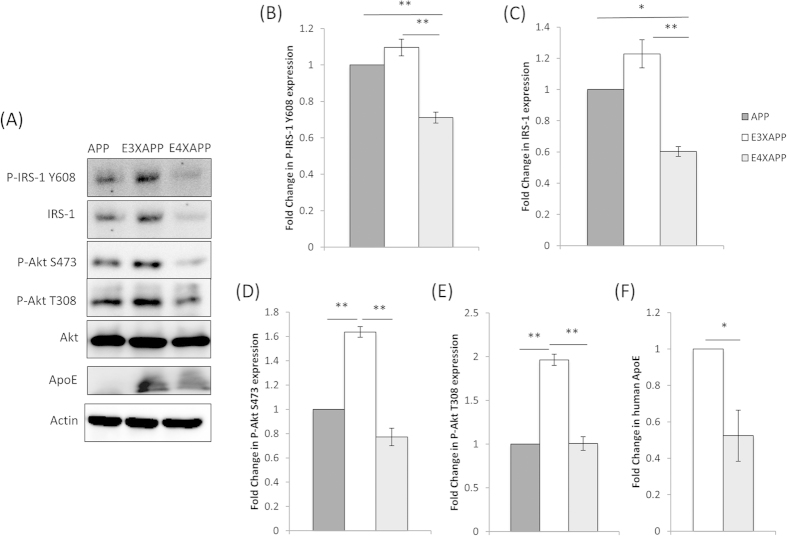
Insulin receptor substrate protein-1 and Akt expression and phosphorylation in 78 week old mouse brain. (**A**) Western blot and (**B**–**F**) densitometric analysis of insulin receptor substrate 1 (IRS-1), phosphorylated IRS-1 (Y608), total Akt, phosphorylated Akt (S473 and T308), and human ApoE levels in the brain of 78 week old APP (dark grey), ApoE3xAPP (E3XAPP, white) and ApoE4xAPP (E4XAPP, grey) mice. β-actin was immunoblotted to ensure similar gel loading of the starting material in each sample. The blot was a representative of six independent experiments. Blot images were cropped for comparison. Densitometry analysis was performed using the NIH ImageJ software and the relative value for ApoE3xAPP and ApoE4xAPP mice was normalized against age-matched APP mice. Each value represents the mean ± SEM for individual mouse brain sample (*n = 6* for each mouse line). One-way ANOVA yielded significant differences between groups (*p < 0.05, **p < 0.01). Student’s t test revealed that human ApoE was also significantly lower in ApoE4xAPP mice as compared to ApoE3xAPP mice (*p < 0.05).

In the aged mouse brain (78 weeks) however, post-hoc Tukey-Kramer analysis showed that IRS-1 expression and phosphorylation at Tyr-608 (Y608, equivalent to Y612 in human IRS-1), one of the two main PI3K p85 binding motifs, was significantly lower in ApoE4xAPP mouse brain as compared to ApoE3xAPP and APP mouse brain (Fig. 2A,B).

IRS-1 expression in ApoE4xAPP brains was ~65% and ~2-fold lower as compared to ApoE3xAPP and APP brains respectively (Fig. 2C, one-way ANOVA IRS-1 F = 5.13). IRS-1 phosphorylation in ApoE4xAPP mouse brain after normalizing for IRS-1 expression was ~54% and ~41% lower than ApoE3xAPP and APP mouse brain (Fig. 2B, one-way ANOVA P-IRS-1 Y608 F = 8.36).

In contrast, brain Akt expression was comparable between APP, ApoE3xAPP and ApoE4xAPP mice (Fig. 2A). However, post-hoc Tukey-Kramer analysis showed that Akt phosphorylation at S473 and T308 were lower in APP and ApoE4xAPP mice as compared to ApoE3xAPP mice. Phospho-Akt at S473 was reduced by ~2-fold and ~63% in ApoE4xAPP and APP mouse brain as compared to ApoE3xAPP mice (Fig. 2D, one-way ANOVA, P-Akt S473, F = 10.51). Similarly, phospho-Akt at T308 was reduced by ~2-fold in ApoE4xAPP and APP mouse brain as compared to ApoE3xAPP mice (Fig. 2E, one-way ANOVA, P-Akt T308, F = 18.16).

At 26 weeks, there was no detectable change in human ApoE levels between ApoE3xAPP and ApoE4xAPP (Figure S2F). However, when the mice aged (at 78 weeks), ApoE protein was reduced by ~50% in ApoE4xAPP as compared to ApoE3xAPP mice (Fig. 2F, t = 3.267, p < 0.05).

### ApoE binding to insulin receptor in ApoE-APP mice

Aβ was reported to bind to the insulin receptor (IR)^43^, where it impaired insulin signaling^20^,^44^ and Aβ clearance^45^. However, these studies were conducted in the absence of human ApoE, and the role of ApoE isoforms in this process was unknown.

**Figure 3:**
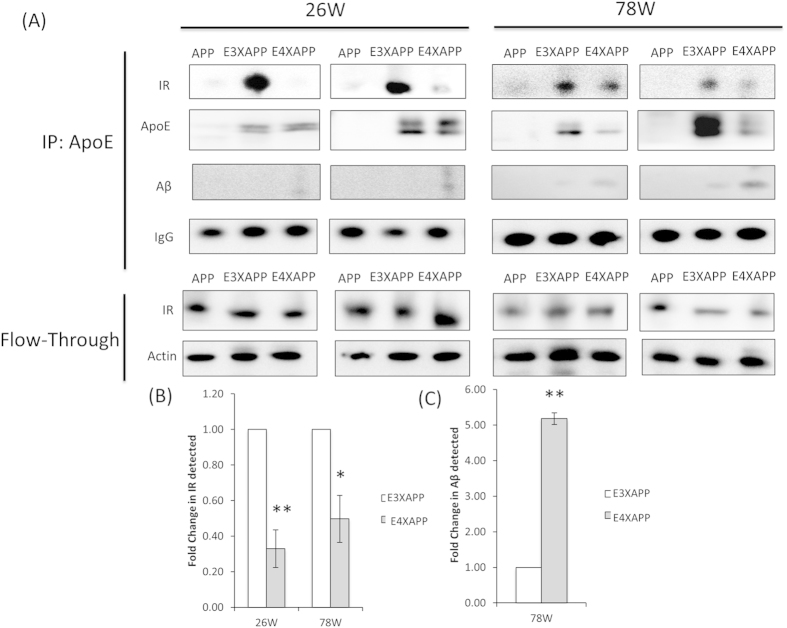
ApoE3 binds to insulin receptor stronger than ApoE4. (**A**) Total brain lysates from 26 (left) and 78 (right) week old APP, ApoE3xAPP (E3XAPP) and ApoE4xAPP (E4XAPP) mice were immunoprecipitated (IP) with anti-ApoE, and were analyzed for human ApoE, insulin receptor (IR), Aβ and mouse IgG. The flow-through refers to the starting material remaining after separation from the agarose beads, and was analyzed for insulin receptor (IR) and β-actin. Each immunoprecipitation for an individual mouse age has been performed on six different mouse brains (n = 6) from each mouse line, and the two blots shown are from two different mouse brain samples. Blot images were cropped for comparison. (**B**,**C**) Densitometry analysis was performed using the NIH ImageJ software and the relative value for ApoE4xAPP mice was normalized against age-matched ApoE3xAPP mice. At 26 and 78 weeks, ApoE4 bound less to IR than ApoE3 in ApoExAPP mice (*p < 0.05, **p < 0.01). At 78 weeks, 5-fold more Aβ binding to ApoE was detected in ApoE4xAPP mice as compared to ApoE3xAPP mice (*p < 0.01).

To determine if ApoE could interact with the insulin signaling proteins, we immunoprecipitated ApoE from 26 and 78 week old brain lysates of our ApoE3xAPP and ApoE4xAPP mice (Fig. 3). We also included the APP mice as a control as it did not express human ApoE. In both the young and aged mouse brain, we detected more IR bound with ApoE3 than IR bound with ApoE4 (26 weeks, t = 3.385, p < 0.01; 78 weeks, t = 2.758, p < 0.05). Similar levels of ApoE were immunoprecipitated with ApoE3 and ApoE4 at 26 weeks. When Aβ levels were increased at 78 weeks, ApoE4 bound more Aβ than ApoE3 (t = 3.599, p < 0.01). This difference was not due to changes in IR expression, as similar IR content was observed in the brain lysates.

As ApoE4xAPP mice had greater Aβ accumulation in the brain at 78 weeks, we investigated if this ApoE association to Aβ and to the insulin receptor was isoform dependent in the presence of increasing Aβ42 concentration. We set up a sandwich ELISA to capture ApoE from our ApoE3 and ApoE4 mice. These ApoE mice did not carry the mutant human APP gene and therefore will not generate human Aβ. After blocking and washing, the wells were incubated with different synthetic Aβ42 concentrations. Unbound Aβ42 were then removed and the wells were probed with either anti-IR or anti-Aβ.

**Figure 4:**
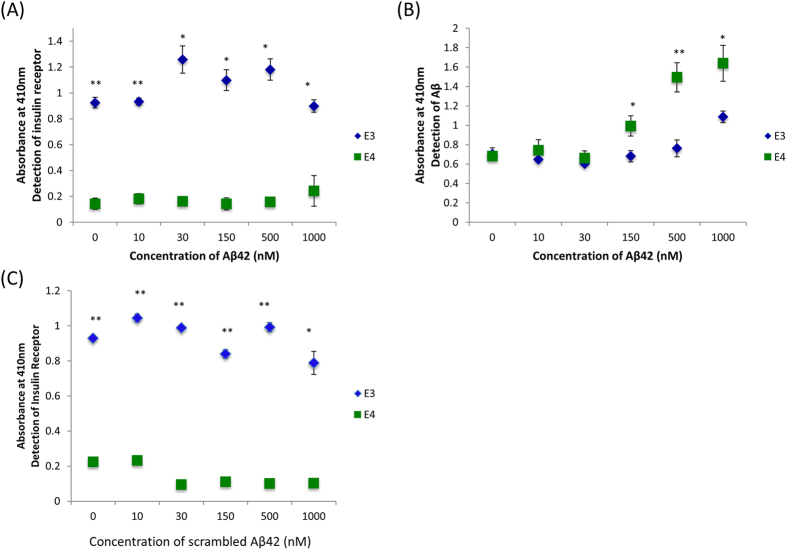
Immunoprecipitation ELISA shows ApoE3 binds to insulin receptor stronger than ApoE4. Total brain lysates from ApoE3 and ApoE4 targeted replacement mice were incubated with increasing concentrations of synthetic (**A,B**) Aβ42, or (**C**) scrambled Aβ42 before adding to ELISA pre-coated with anti-ApoE. The captured protein complexes were analyzed for insulin receptor (IR) in (**A**) and (**C**), and for Aβ in (**B**). Each value represents the mean ± SEM for individual mouse brain sample. Statistical comparison was made between ApoE3 and ApoE4 mouse brain sample at each Aβ42 concentration (*p < 0.05; **p < 0.001, using Student’s t-test; n = 4). The t-values for each comparison are shown in table S1.

As shown in Fig. 4A (Table S1), the level of IR bound to the captured ApoE3 was not significantly altered with increasing Aβ42 concentrations. On the other hand, ApoE3 and ApoE4 had similar abilities to associate with Aβ42 between 0–30 nM. However, ApoE4 associated with more Aβ42 as the concentrations increased beyond 30nM (Fig. 4B, Table S1). Incubation with scrambled Aβ did not affect ApoE3 binding to IR (Fig. 4C, Table S1).

### ApoE4 and Aβ peptide impair insulin signaling in hippocampal neurons

Next, we wanted to know if ApoE4 expression was sufficient to cause insulin signaling impairment and whether the insulin signaling deficit was a result of the co-presence of ApoE4 and Aβ42.

**Figure 5:**
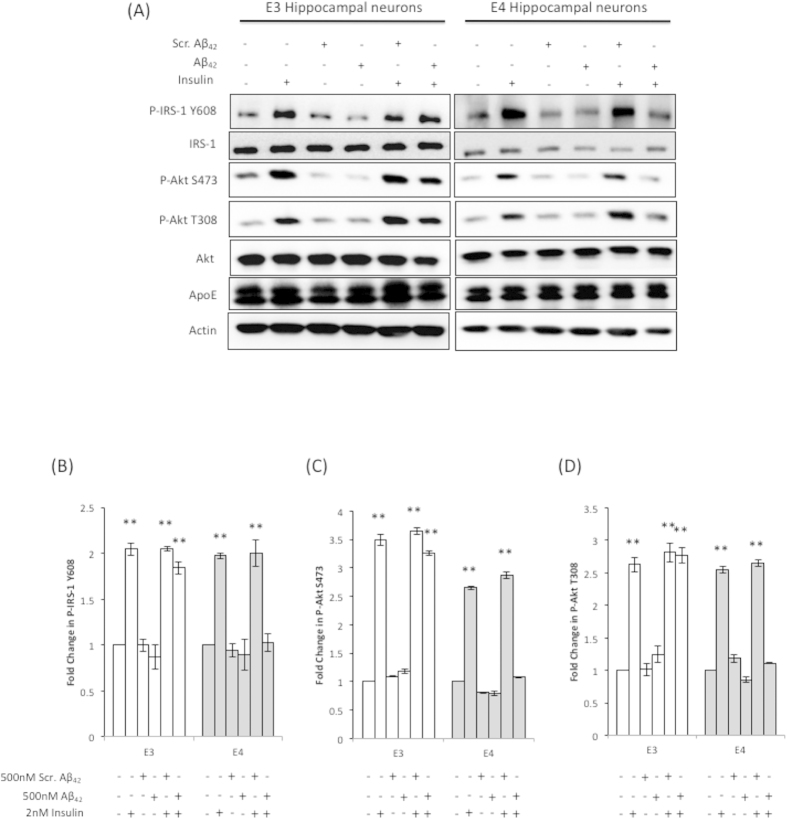
Aβ42 lowers IRS-1 and Akt phosphorylation in ApoE4 hippocampal neurons. (**A**) Western and (**B**–**D**) densitometric analysis of the effects of insulin treatment (2 nM) on ApoE expression and, IRS-1 and Akt expression and phosphorylation in ApoE3 (E3) and ApoE4 (E4) hippocampal neurons exposed to 500 nM of Aβ42 or scrambled Aβ42. β-actin was immunoblotted to ensure similar gel loading of the starting material in each sample. The blot shown was one of three experiments using different primary hippocampal cultures. Blot images were cropped for comparison. Each value represents the mean ± SEM and the relative value for each treatment was normalized against no treatment (NT) of the same hippocampal cell line. One-way ANOVA revealed significant differences in P-IRS-1 Y608, P-Akt S473 and P-Akt T308 expression among treatments within each cell line (**p < 0.01).

We used DIV10 hippocampal neurons cultured from ApoE3 and ApoE4 mice. We decided to use these matured neurons to test the Aβ42 susceptibility in the presence of different ApoE isoforms because AD is an ageing disease and immature neurons were more resistant to neurotoxic insults^46^,^47^,^48^. Insulin treatment (2 nM) activated IRS-1 (Y608) and Akt (S473 and T308) phosphorylation in both cultures (Fig. 5). However, 500 nM Aβ42 or scrambled Aβ42 peptides added to ApoE3 and ApoE4 hippocampal neurons had no effect on IRS-1 and Akt expression and phosphorylation, similar to non-treated neurons. The Aβ42 concentration used in our study is comparable to other studies used on primary neurons^44^,^49^. In ApoE3 hippocampal neurons, F = 22.98, 16.26, 8.22, (p < 0.01) for P-IRS-1 Y608, P-Akt S473 and P-Akt T308 respectively. In ApoE4 hippocampal neurons, F = 8.27, 17.40, 16.65 (p < 0.01) for P-IRS-1 Y608, P-Akt S473 and P-Akt T308 respectively.

We next proceeded to determine the effect of insulin on neurons pre-treated with Aβ42 or scrambled Aβ42 peptides. As shown in Fig. 5, insulin activated IRS-1 and Akt phosphorylation in both ApoE3 and ApoE4 hippocampal neurons pre-treated with scrambled Aβ42 peptides. However, post-hoc Tukey-Kramer analysis showed that insulin only activated IRS-1 and Akt phosphorylation in ApoE3 neurons pre-treated with Aβ42 peptides. Insulin had no effect on IRS-1 and Akt phosphorylation in ApoE4 hippocampal neurons pre-treated with Aβ42 peptides.

In Fig. 5, we also noted that insulin and/or Aβ42 treatment have no effect on ApoE level. Similar to the mouse brain (Figure S1), there were two ApoE bands; the native 34kDa protein and the higher molecular weight sialylated ApoE isoprotein^42^. Sialylated ApoE isoproteins were reported to associate with neurons in human ApoE transgenic mice and in human brain, and they contribute significantly (~60%) to the total neuronal ApoE level^42^. Therefore, the high level of the higher molecular weight ApoE protein band detected in our ApoE3 and ApoE4 neuronal cultures suggest that the detected ApoE is predominantly derived from neurons.

Together, these results showed that insulin signaling impairment in the presence of Aβ42 was isoform specific, where the expression of ApoE4 reduced response to insulin stimulation and the expression of ApoE3 prevented Aβ42 induced insulin signaling deficits in neurons.

## Discussion

The ApoE4 allele is the strongest genetic risk factor for AD^3^. While its mechanism is still currently unknown, it is certain that ApoE4 expression leads to earlier onset of the disease and rapid accumulation of Aβ plaques. Our results showed that ApoE4 and Aβ co-expression resulted in the impairment of the insulin signaling pathway. In young mice, where Aβ levels were lower, the expression of proteins within the insulin signaling pathway remained comparable among APP, ApoE3xAPP and ApoE4xAPP mice brains. This suggests that insulin signaling impairment was a downstream effect of Aβ plaque accumulation. When the mice aged, levels of proteins within the insulin signaling pathway were markedly reduced in APP and ApoE4xAPP mice brains.

Although previous studies reported that high Aβ levels led to insulin signaling impairment, these experiments were not done in the presence of human ApoE^20^. Our data showed that ApoE genotypes could modulate Aβ-mediated insulin signaling impairment. This predicts that ApoE4 carriers may experience insulin signaling impairment at a much earlier stage than ApoE3 carriers, albeit insulin signaling in both ApoE3 and ApoE4 carriers will eventually become similarly impaired in end stage post-mortem AD tissues^10^.

As ApoE4xAPP mice expressed higher Aβ42/40 ratios than ApoE3xAPP mice, we used primary hippocampal neurons to investigate if the more impaired insulin signaling in ApoE4xAPP mice was a result of more Aβ42 being produced, or if it was an inherent result of ApoE4 by itself. Interestingly, the presence of ApoE4 alone did not lead to a defective response to physiological levels of insulin, and the response of ApoE4 hippocampal neurons to insulin alone was comparable to the response of ApoE3 hippocampal neurons. However, in the presence of Aβ42, physiological levels of insulin failed to elicit a downstream response in ApoE4 hippocampal neurons. In contrast, ApoE3 hippocampal neurons remained sensitive to 2 nM insulin, despite the presence of Aβ42. Our data demonstrated that the presence of both ApoE4 and Aβ42 led to insulin signaling impairment in hippocampal neurons, and we asked what could be the possible difference in the nature of ApoE3 and ApoE4 that might account for this.

Although differential binding between ApoE isoforms and Aβ were reported, the results were inconsistent^50^,^51^,^52^,^53^. This could be due to different ApoE lipidation states^50^,^52^,^53^, detection methods, and different mouse lines^53^. Nevertheless, this differential ApoE-Aβ binding could influence Aβ pathogenic effects^54^,^55^.

While Aβ had been shown to bind to the insulin receptor^43^, ApoE isoforms interactions with the IR were not studied. Here, we showed that ApoE3 had a greater association with the insulin receptor at every age, regardless of Aβ42 concentration. In contrast, ApoE4 had a weaker association with the insulin receptor, independent of age and Aβ42 levels.

Using ApoE3 and ApoE4 targeted replacement mice, we showed that even with the same levels of Aβ42, ApoE4 still had a much lower capacity to associate with the insulin receptor, further confirming our mouse brain immunoprecipitation data. However, at 78 weeks, we observed a slight increase in ApoE4 association with the insulin receptor. This slight increase in the insulin receptor bound might not due to ApoE4 associating with the insulin receptor, but the net effect of Aβ42 binding to the insulin receptor, which was detected while being pulled down^44^.

In summary, this study suggested that the ApoE4 genotype could lead to an earlier impairment of brain insulin signaling, possibly contributing to an earlier onset of AD. Our results also showed that this ApoE isoform dependent effect on brain insulin sensitivity was modulated by the difference in ability of the ApoE isoforms to associate with the insulin receptor.

## Materials and Methods

### Animals

Experiments involving the use of animals and primary hippocampal neuron cultures were carried out in accordance with protocols R13–4468 and BR 13–4458 approved by the Institutional Animal Care and Use Committees (IACUC) at the National University of Singapore. The ApoE3xAPP and ApoE4xAPP mice were generated by crossing the human ApoE3 and ApoE4 targeted replacement mice^21^, with the APP J20 transgenic mice^23^. The APP J20 mice carried a mutant human APP gene bearing the Swedish (K670N/M671L) and Indiana (V717F) mutations. All mice in the study were bred and housed conventionally, under ambient conditions (12hrs dark, 12hrs light). They were kept on 2018 Teklad Global 18% Protein Rodent Diet (Harland Laboratories). All experiments were performed on female APP, ApoE3xAPP and ApoE4xAPP mice at 26 and 78 weeks of age.

### Preparation of brain homogenates

Mouse brain homogenates were prepared as described in our earlier study^56^. Briefly, mice brain tissues were snap frozen in liquid nitrogen when harvested and the wet weight of the tissues (in mg) was determined using an electronic balance. The entire left hemisphere of the brain was digested with ice-cold 1x RIPA lysis buffer (Cell Signaling Technology) containing detergents (1% Nonidet P40 and 1% sodium deoxycholate) together with the protease inhibitor cocktail tablet (Roche). Lysates were homogenized using a hand held motorized pestle (Sigma-Aldrich) on ice. Tissue lysates were collected for protein quantification using BCA analysis (ThermoFischer Scientific)

### Aβ ELISA

Preparation of brain samples for Aβ ELISA was performed as follows. Whole brain lysates were diluted 500-fold in ice-cold 5 M guanidine hydrochloride, pH 8, and incubated at room temperature with vigorous shaking at 450 rpm for 3 hours. Next, the samples were further diluted 100-fold in EIA buffer, provided by the manufacturer. Quantification of Aβ42/40 ratios were performed according to manufacturer’s instructions using the Human Amyloid-β (1–40) (N) Assay Kit and the Human Amyloid-β (1–42) (N) Assay Kit (Immuno-Biological Laboratories Co.).

### Western Blot

Western blot was performed as previously described^56^, and run using a 10% Tris-glycine polyacrylamide gel or NuPAGE® Bis-Tris gels (Life Technologies). After the proteins were transferred onto a nitrocellulose membrane, the membranes were probed with the following primary antibodies and exposed to horseradish peroxidase (HRP)-conjugated secondary antibodies. The brands were visualized by chemiluminescence on the Image Station 4000R (Carestream Health Inc).

The primary antibodies used in this study were anti-human ApoE (Calbiochem, Cat#178479), anti-human ApoE (Santa Cruz, Cat#SC98573), anti-P-IRS-1 (Y608) (Millipore, Cat#09-432), anti-IRS-1 (Cell Signal Tech, Cat#2382), anti-IR (Cell Signal Tech, Cat#3020), anti-P-Akt (S473) (Cell Signal Tech, Cat#4060), anti-P-Akt (T308) (Cell Signal Tech, Cat#2965), anti-Akt (Cell Signal Tech, Cat#4691), anti-Actin (Sigma, Cat#A2066), and anti-Aβ (1–17) (6E10) (Covance, Cat#SIG-39300). Densitometry analysis of the bands was performed as described^57^ by measuring the optical densities of the targeted protein bands relative to the β-actin level from the same brain sample. For protein phosphorylation, the optical densities of the phosphorylated protein bands were measured relative to the targeted total protein level from the same brain sample. The analysis was performed using the NIH ImageJ software.

### Immunoprecipitation

Brain lysates were pre-cleared for 1hr at room temperature before incubating with anti-human ApoE (Santa Cruz, Cat#SC98573) overnight on a rotating rotor at 4 ^o^C. Pierce Protein A/G Plus Agarose beads (Research Instruments Co. Ltd.) were added and the lysates were rotated on a rotating rotor for 4 h at room temperature. Subsequently, the beads and lysates were washed 5 times with PBS and prepared for immunoblot analysis as described above.

### Aβ42 preparation

Lyophilized Aβ42 and scrambled Aβ42 (1^st^ Base) were purchased and stored at −80 ^o^C. Aβ42 and scrambled Aβ42 used in the experiments were dissolved in DMSO to yield a concentration of 500 μM, and immediately diluted in PBS or culture medium for use.

### Immunoprecipitation ELISA

Brain lysates were incubated with varying concentrations of Aβ42 overnight on a rotating rotor at 4 ^o^C. To capture ApoE, Nunc-Immuno^™^ MicroWell^™^ 96 well solid plates (Sigma Aldrich, Singapore) were coated with anti-human ApoE (Santa Cruz, Cat#SC98573) overnight, and blocked with 10% FCS the following day. The incubated brain samples are loaded onto the plates and incubated overnight. The following day, the unbound brain samples were removed. The plates were then washed before incubating with either anti-IR (Cell Signal Tech, Cat#3020), or anti-Aβ (1–17) (6E10) (Covance, Cat#SIG-39300) for 1h. The plates were washed again before adding in mouse horseradish peroxidase (HRP)-conjugated secondary antibodies. After 1h, 2,2′-azino-bis(3-ethylbenzothiazoline-6-sulphonic acid) (ABTS) solution was added and incubated for 20 minutes. The absorbance from the wells was then read at absorbance of 410 nm.

### Acute Insulin treatment of Hippocampal neurons

Hippocampal neurons were obtained from dissection of P0 pups of either ApoE3 or ApoE4 mice as previously described^58^. ApoE3 or ApoE4 neurons at DIV10 were incubated with either 500 nM of Aβ42 or scrambled Aβ42 24 h prior to starvation. The Aβ42 concentration used in this study was comparable to other studies using primary neurons^44^,^53^. On the day of the experiment, the Neurobasal media (Life Technologies) was removed from the wells and the neurons were starved in Earle’s Balanced Salt Solution (EBSS) (Life Technologies) for 2 hours. After starvation, 2 nM of insulin (Sigma Aldrich) was added into the respective wells and incubated for 30 minutes. Following the acute insulin treatment, the neurons were collected for Western Blot analysis.

### Statistical analysis

When comparing between two groups (ApoE3xAPP and ApoE4xAPP), statistical significance was calculated using two-tailed Student’s T-test, as described previously^56^. One-way ANOVA test, followed by post-hoc Tukey-Kramer test was used to compare differences among groups (between APP, ApoE3xAPP and ApoE4xAPP, and between hippocampal neuron treatment groups). P values less than 0.05 were considered to be statistically significant.

## Additional Information

**How to cite this article**: Chan, E. S. *et al.* Differential interaction of Apolipoprotein-E isoforms with insulin receptors modulates brain insulin signaling in mutant human amyloid precursor protein transgenic mice. *Sci. Rep.* **5**, 13842; doi: 10.1038/srep13842 (2015).

## Supplementary Material

Supplementary Information
