# Supplementary material for: Differential interaction of Apolipoprotein-E isoforms with insulin receptors modulates brain insulin signaling in mutant human amyloid precursor protein transgenic mice
Source: Sci Rep. 2015 Sep 8;5:13842. doi: 10.1038/srep13842 (PMC4561911; doi:10.1038/srep13842)
Supplement: Supplementary Information [file srep13842-s1.pdf]

Supplementary Information

Differential interaction of Apolipoprotein-E isoforms with insulin receptors modulates brain insulin signaling in mutant human amyloid precursor protein transgenic mice.

Elizabeth S Chan [1], Christopher Chen [2, 3], Gregory M Cole [4, 5] and Boon-Seng Wong [1, +]

Departments of Physiology [1] and Pharmacology [2], Yong Loo Lin School of Medicine, National University of Singapore, Singapore.

[3] Memory Ageing and Cognition Centre (MACC), National University Health System (NUHS), Singapore.

[4] Departments of Medicine and Neurology, University of California, Los Angeles, USA.

[5] Geriatric Research and Clinical Center, Greater Los Angeles Veterans Affairs Healthcare System, Veterans Affairs Medical Center, Los Angeles, California, USA.

[+] Correspondence to bswong@nus.edu.sg

Figure S1. ApoE expression in the ageing ApoExAPP mice. Western blot analysis of human ApoE expression in the brain of APP, ApoE3xAPP (E3XAPP) and ApoE4xAPP (E4XAPP) mice at 26 and 78 weeks old. β-actin was immunoblotted to ensure similar gel loading of the starting material in each sample. The blot was a representative of four experiments using different mouse brain samples (n=4). Blot images were cropped for comparison.

Figure S2. Insulin receptor substrate protein-1 and Akt expression and phosphorylation in 26 week old mouse brain. (A) Western blot and (B-F) densitometric analysis of insulin receptor substrate 1 (IRS-1), phosphorylated IRS-1 (Y608), total Akt, phosphorylated Akt (S473 and T308), and human ApoE levels in the brain of 26 week old APP (dark grey), ApoE3xAPP (E3XAPP, white) and ApoE4xAPP (E4XAPP, grey) mice. β-actin was immunoblotted to ensure similar gel loading of the starting material in each sample. The blot was a representative of six independent experiments. Blot images were cropped for comparison. Densitometry analysis was performed using the NIH ImageJ software and the relative value for ApoE3xAPP and ApoE4xAPP mice was normalized against age-matched APP mice. Each value represents the mean ± SEM for individual mouse brain sample ($n=6$ for each mouse line). One-way ANOVA showed no significant change in protein expression and phosphorylation between the three mouse lines.

Table S1. Statistical analysis of immunoprecitation ELISA between ApoE3 and ApoE4 mouse brain at different Aβ42 concentrations (Figure 4). ApoE3 binds more to IR than ApoE4 at all concentrations of Aβ42.

Detection of IR (Figure 4A)

Conc. of Aβ42	t-value
0	3.365
10	4.330
30	2.750
150	3.210
500	2.784
1000	2.636

Detection of Aβ (Figure 4B)

Conc. of Aβ42	t-value
0	0.2470
10	1.089
30	0.7353
150	2.653
500	4.327
1000	2.292

Detection of IR (Figure 4C)

Conc. of Scr. Aβ42	t-value
0	2.983
10	3.292
30	6.050
150	3.079
500	4.094
1000	2.465

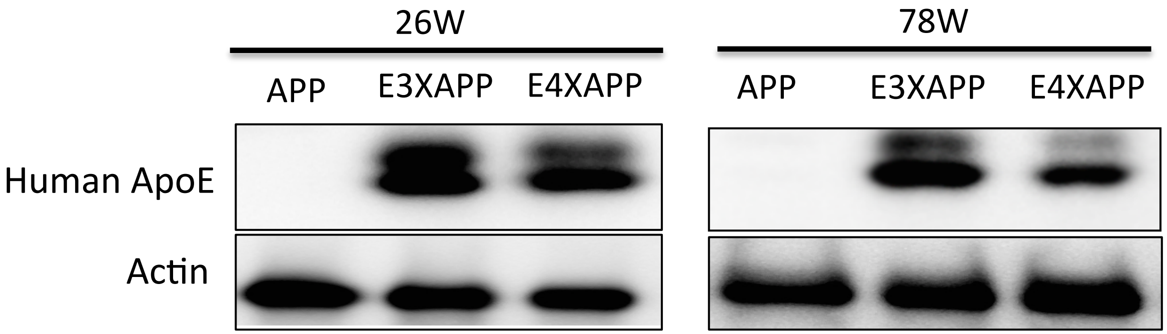

Figure S1 (Chan et al)

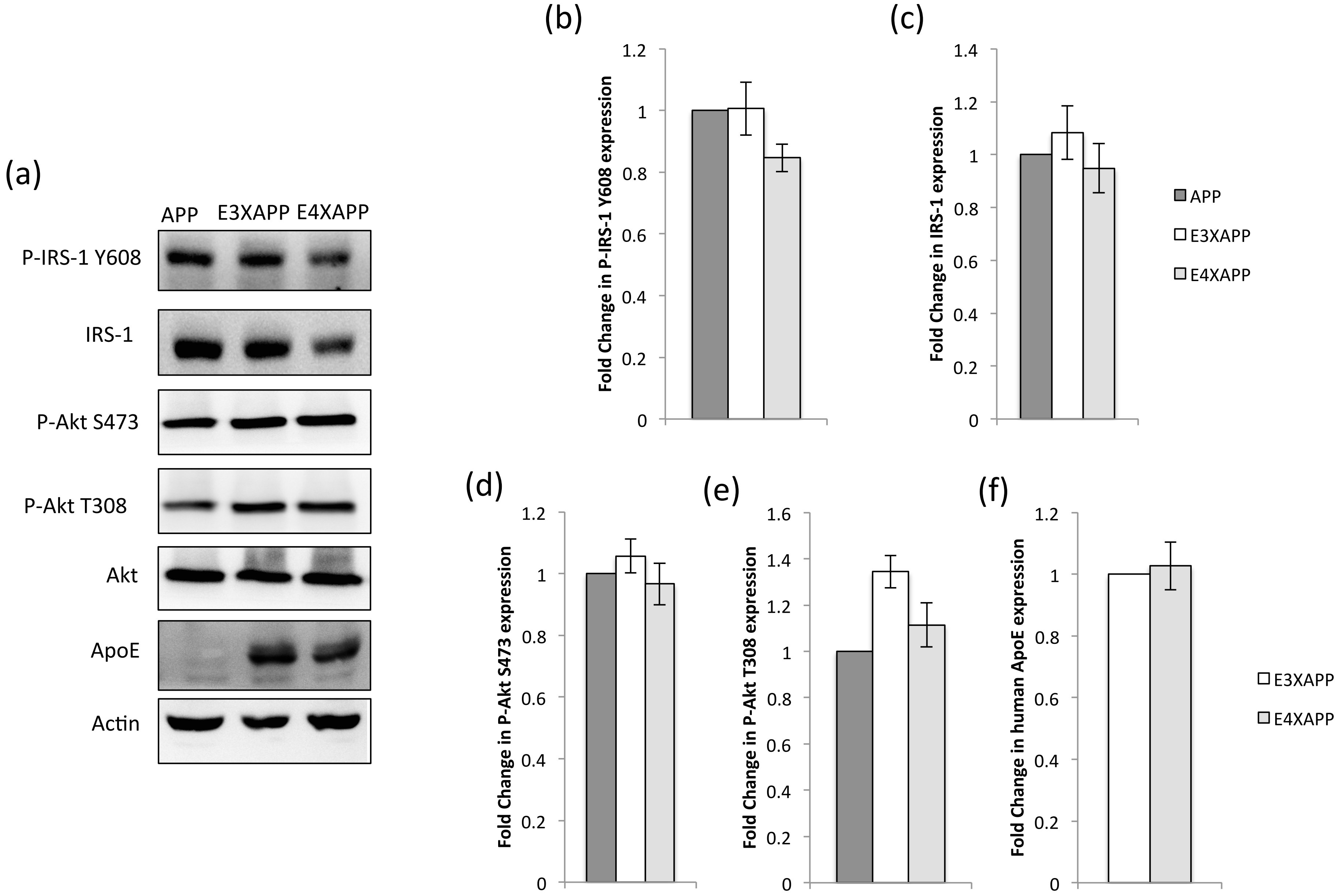

Figure S2 (Chan et al)
